# Recent Advances in Rapid and Highly Sensitive Detection of Proteins and Specific DNA Sequences Using a Magnetic Modulation Biosensing System

**DOI:** 10.3390/s22124497

**Published:** 2022-06-14

**Authors:** Shira Roth, Michael Margulis, Amos Danielli

**Affiliations:** Faculty of Engineering, The Institute of Nanotechnology and Advanced Materials, Bar-Ilan University, Max and Anna Webb Street, Ramat Gan 5290002, Israel; shirakoren111@gmail.com (S.R.); margulis@gmail.com (M.M.)

**Keywords:** magnetic beads, biosensing, nucleic acids, serology, protein-protein interactions, magnetic modulation biosensing

## Abstract

In early disease stages, biomolecules of interest exist in very low concentrations, presenting a significant challenge for analytical devices and methods. Here, we provide a comprehensive overview of an innovative optical biosensing technology, termed magnetic modulation biosensing (MMB), its biomedical applications, and its ongoing development. In MMB, magnetic beads are attached to fluorescently labeled target molecules. A controlled magnetic force aggregates the magnetic beads and transports them in and out of an excitation laser beam, generating a periodic fluorescent signal that is detected and demodulated. MMB applications include rapid and highly sensitive detection of specific nucleic acid sequences, antibodies, proteins, and protein interactions. Compared with other established analytical methodologies, MMB provides improved sensitivity, shorter processing time, and simpler protocols.

## 1. Introduction

Due to their high sensitivity [1,2,3], ease of operation, and multiplexing capabilities [4,5,6], fluorescence-based immunoassays are widely used in many in vitro diagnostic (IVD) devices. To capture a target molecule, a typical fluorescence-based assay uses capture probes, such as an antibody, an oligonucleotide or an antigen, that are immobilized to a capture surface, such as a 96-well plate [7] or magnetic beads [8,9,10]. The target molecules are also tagged by fluorescently labeled probes, which can be detected by an optical reader.

However, when the analyte of interest is tagged with a fluorescent probe, at low concentrations of the target molecule, the resulting signal is very weak and commonly obscured by the background noise [11]. The primary sources of background noise in fluorescence-based assays include red-shifted photons, originating from Raman scattering of the solvent, residual fluorescence from unbound fluorescent molecules, and autofluorescence of the capture surfaces.

The primary approach to removing residual fluorescence from unbound fluorescent molecules is to incorporate washing and separation steps in the assay [12]. For example, the well-established Luminex xMAP technology uses magnetic beads to capture and detect a wide range of target molecules. In an xMAP-based device, such as the Luminex 200, Flexmap 3D (Luminex Corp, Austin, TX, USA), or the Bio-Plex 200 (Bio-Rad Laboratories, Hercules, CA, USA), antibody-conjugated magnetic beads first capture the target analytes and then fluorescently labeled antibodies are used to detect them. By examining individual beads one at a time, this highly sensitive technology allows simultaneous detection of multiple targets in the same sample well. However, similar to conventional enzyme-linked immunosorbent assays (ELISA), xMAP-based assays require multiple washing steps to remove the unbound fluorophores, adding time and labor to the testing protocols. Moreover, because of the size, cost, and complexity of these flow cytometry-based detection devices, they are usually found only in high-resource central labs.

As an alternative, background fluorescence from unbound fluorescent molecules can be reduced by using modulation. For example, magnetically modulated optical nanoprobes (MagMOONs) [13,14] are micro- and nanoparticles with one hemisphere functionalized with antibodies or a molecular recognition element. Subsequent labeling of the captured antigen with a fluorescent tag in a sandwich assay increases the brightness of this hemisphere. Thus, these particles blink in response to rotating magnetic fields, enabling background subtraction of the fluorescence signal originating from the unmodulated background [13]. Modulation of the MagMOONs signals enhances sensitivity and reduces the need for the washing and separation steps commonly used in other heterogeneous assays [14]. However, to achieve high modulation depth, the signal ratio between the fluorescently covered half of the particle and the naked half has to be high, thereby complicating the production of these particles and limiting their use to applications that tolerate a high number of fluorescent molecules per bead.

Here, we describe an alternative fluorescence-based technology, termed magnetic modulation biosensing (MMB), that also uses modulation of magnetic beads to reduce background noise but concentrates them to increase the signal. First, we describe the basic principles of the MMB system, then we review the analytical and clinical performance of the system in various applications. Finally, we present the latest modifications of the system, namely magnetically aggregated biosensors (MAB) and optical modulation biosensing (OMB).

## 2. Magnetic Modulation Biosensing (MMB) Technology Principles

**Figure 1:**
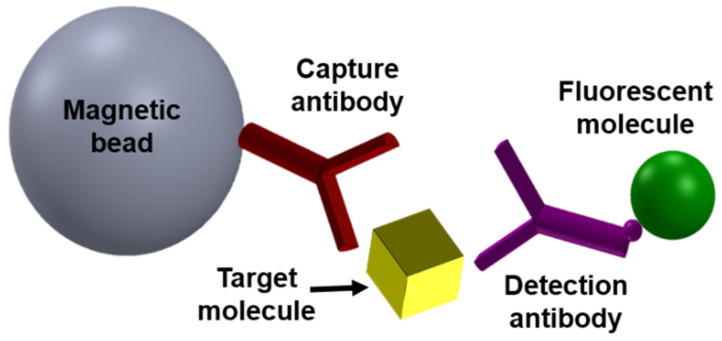
Schematic of a typical sandwich immunoassay. A typical fluorescence-based “sandwich” assay, showing a magnetic bead coated with a capture antibody, a target molecule (e.g., human interleukin 8), and a detection antibody conjugated to a fluorescent molecule (e.g., phycoerythrin). Reprinted from [15], with the permission of AIP Publishing 2019.

MMB technology was developed to address the low signal-to-noise ratio problem that afflicts most of today’s fluorescence-based assays at low analyte concentrations. Similar to other fluorescence-based detection methods, in MMB the target analyte is attached to a capture surface (in this case, a magnetic bead) and tagged with a fluorescently labeled probe to form a “sandwich” assay (Figure 1). To detect the concentration of the target analyte, an external alternating magnetic field aggregates the beads with the attached target molecules from the entire sample volume into the detection area, where they are moved in and out of an orthogonal laser beam [11].

Aggregating the beads with the attached targets from the entire volume allows working with higher sample volumes, further improving the sensitivity of MMB-based assays [10]. Moving the bead cluster from side to side separates the signal from the background noise of the non-magnetized solution, and thereby eliminates the need for numerous washing steps, shortens the detection time, and simplifies the operation.

**Figure 2:**
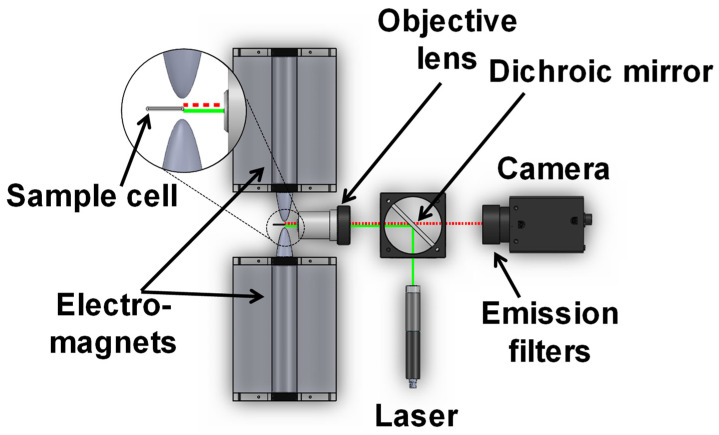
Schematic of a magnetic modulation biosensing (MMB) system.

Briefly, the MMB system (Figure 2) uses a 532 nm laser diode module working at 0.25 mW [11,16]. The excitation laser light is reflected by a dichroic mirror and reshaped and focused by an objective lens to provide a ~150 µm diameter beam on a borosilicate cuvette containing the sample. The emitted fluorescence is collected by the same optical system and detected by a digital camera equipped with two identical optical emission filters. Two electromagnets on opposite sides of the sample cell apply an alternating magnetic field gradient with a modulation frequency of 1 Hz, which aggregates the beads and transports them in a periodic lateral motion [17]. As the beads with the attached fluorescently labeled probes and target analyte pass in front of the tightly focused laser beam, the resulting emitted fluorescence creates a flashing signal that is easily distinguished from the constant background of the unbound fluorescent molecules. A total of 600 images are acquired within 12 s, at a rate of 50 frames per second. The mean grey value from the laser-illuminated area of each image is calculated, and the peak-to-peak differences over time are averaged.

This highly sensitive MMB technology has been demonstrated in several research [11,16] and clinical [16,18,19] applications. For example, the MMB system was used to detect ~0.08 ng/L of the human protein Interluekin-8 (IL-8) in plasma [11], ~100 ng/L of anti-Zika virus IgM and IgG antibodies in serum [16], and fM concentrations of specific DNA sequences [18].

## 3. Technology Applications

### 3.1. Detection of Protein Biomarkers

#### 3.1.1. Serological Assays

Viral infections can be diagnosed either directly, by detecting the viral components (i.e., nucleic acids or proteins) using molecular or antigenemia assays, or indirectly, by detecting an immune response agent (e.g., IgM or IgG antibodies) [20]. Direct diagnostic tests, such as RT-qPCR for coronavirus detection, are highly specific and accurate, and positive results indicate the presence of an acute-phase infection. Nevertheless, due to the relatively short time during which the virus resides in body fluids, the applicability of these tests is limited [20]. In addition, they are laborious and are not useful in point-of-care settings. Serological tests include the enzyme-linked immunosorbent assay (ELISA) [21,22] and plaque-reduction neutralization tests [21]. Because they detect antibodies produced by the immune response, these tests can be performed long after the virus has disappeared from the body. However, they lack specificity [23] and clinical sensitivity [22], exhibit high cross-reactivity [24], and have lengthy protocols.

**Figure 3:**
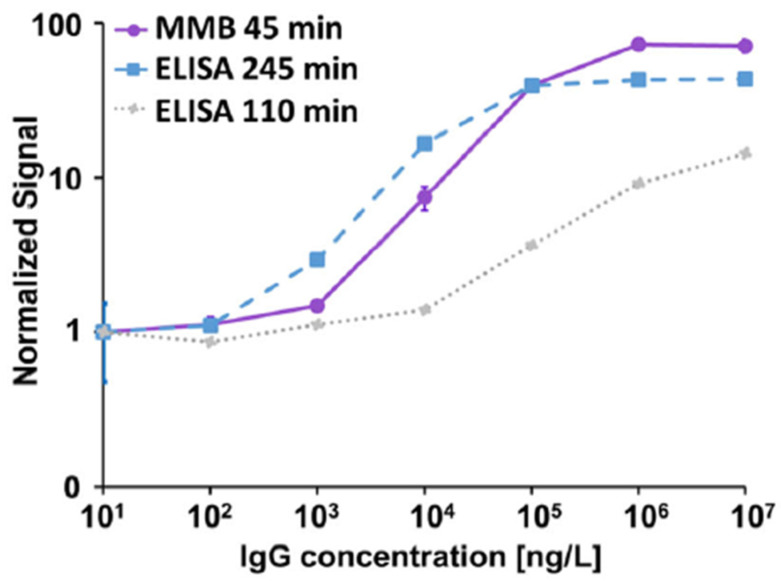
Dose response comparison between the 45-min MMB-based assay and the 110- and 245-min ELISA tests. The calculated LoD of the MMB test is 129 ng/L. The calculated LoDs of the ELISA tests are 6267 and 817 ng/L, respectively. Reprinted from [25] with the permission of MDPI 2022.

Compared with the gold standard ELISA, the MMB-based Zika Virus (ZIKV) and SARS-CoV-2 serological assays have much better analytical performance. For example, in a recent study, the MMB ZIKV serological assay detected as little as 7×10^3^ and 9×10^3^ ng/L of anti-ZIKV IgM and IgG antibodies, respectively, concentrations that are ~85- and ~5-fold lower than the concentrations detected by EUROIMMUN ELISA [20]. Compared with ELISA, the MMB SARS-CoV-2 serological assay had a ~6-fold lower LoD using recombinant anti-SARS-CoV-2 IG antibody (129 ng/L vs. 817 ng/L) (Figure 3), and a ~3.8-fold lower LoD using the WHO international standard recombinant human IgG anti-SARS-CoV-2 S1 antibody (1.14 IU/mL vs. 4.35 IU/mL) [25].

**Figure 4:**
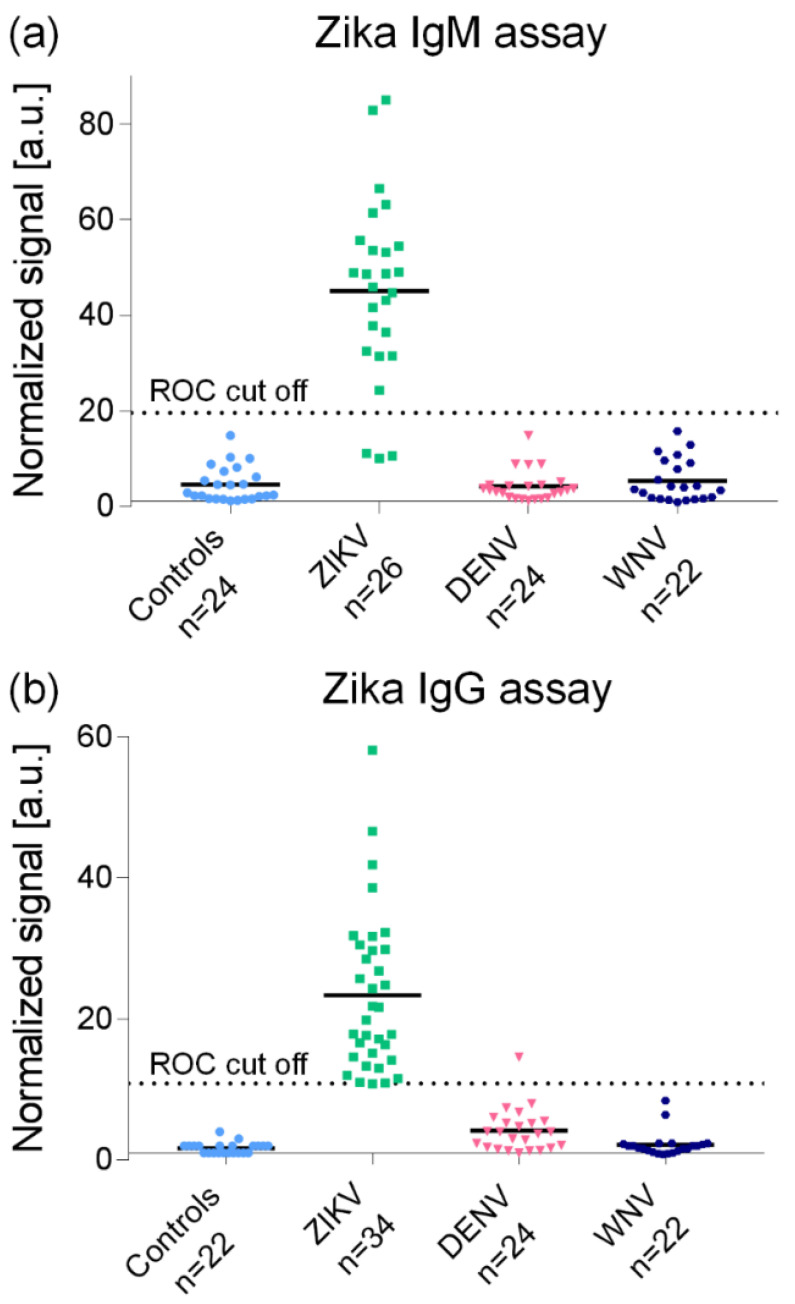
Sensitivity, specificity, and cross-reactivity of the MMB Zika IgM and IgG serological assays. (**a**) Zika IgM and (**b**) Zika IgG assay results. The Controls (blue) were taken from healthy patients. The DENV (red) and WNV (black) are Dengue virus (DENV)- and West Nile virus (WNV)-positive samples were taken from enzyme-linked immunosorbent assay-positive patients. All samples were obtained from the National Center for Zoonotic Viruses at the Central Virology Laboratory of the Ministry of Health at Sheba Medical Center, Israel. The ZIKV (green) are Zika Virus positive samples taken from quantitative reverse-transcription polymerase chain reaction/neutralization-positive patients. The IgM and IgG samples were obtained on days 1–60 and 7–180 post-symptom onset, respectively. The Zika-positive samples were collected from Israeli travelers presenting at the Institute of Tropical Medicine at Sheba Medical Center after returning from Zika-endemic areas. The MMB IgM assay detected 23 of 26 Zika-positive samples (88% sensitivity), and the MMB IgG assay detected 33 of 34 Zika-positive samples (97% sensitivity) The specificity of the MMB assay for both IgM and IgG was 100%. The cross-reactivity to WNV was 0% for both IgM and IgG. The cross-reactivity to DENV was 0% for IgM and 4% for IgG. Reprinted from [20] by permission of Oxford University Press 2018.

In terms of clinical sensitivity and specificity, the MMB-based anti-SARS-CoV-2 IgG assay had similar sensitivity (93% vs. 92%) and specificity (98% vs. 99%) to ELISA, but the assay’s turnaround time was much faster (45 min. vs. 245 min.) (Figure 3). The MMB-based anti-ZIKV IgM and IgG serological assays (Figure 4) exhibited much higher clinical sensitivities (88% and 97%) than EUROIMMUN ELISA (38% and 74%) [20], and the specificities were 100%. The cross-reactivities of the MMB-based anti-ZIKV IgM and IgG assays to other viruses of the *Flavivirus* genus, such as WNV and DENV, were 0% and 4%, respectively (Figure 4).

Another important aspect of serological assays is their detection window time (i.e., how early or how late the assay can detect antibodies). Compared with EUROIMMUN ELISA, the MMB-based anti-ZIKV IgM assay had a much broader detection window for IgM antibodies (5–180 vs. 7–29 days post symptom onset) [20]. Compared with ELISA, the MMB-based anti-SARS-CoV-2 IgG assay identified individuals as positive following vaccination with BNT162b2 mRNA much earlier (on day 7 days vs. day 14).

Finally, compared with EUROIMMUN ELISA, the MMB-based anti-ZIKV serological assays had a lower incidence of false-positive results. Eleven serum samples that were negative for RT-qPCR and neutralization tests, but positive in the EUROIMMUN IgM or IgG assays, were tested by the MMB-based assays. Ten out of the eleven samples were identified as negative by the MMB system. Only one sample was falsely identified as positive [20]. Similarly, compared with ELISA, the MMB-based anti-SARS-CoV-2 IgG assay had a lower incidence of false-negative results. Out of 25 RT-qPCR SARS-CoV-2-positive samples from a large cohort study, the MMB system was able to correctly identify 14 samples (56%) that were identified as negative or borderline using the ELISA test.

#### 3.1.2. Detection of Interleukin-8 (IL-8) in Plasma

**Figure 5:**
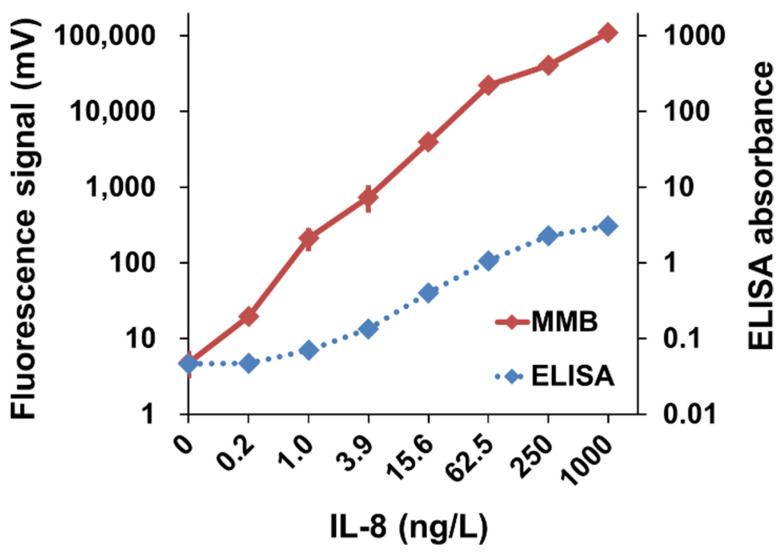
IL-8 dose response. Comparison of IL-8 dose response in 25% plasma with MMB and a standard ELISA kit. Reprinted from [11], Copyright (2017), with permission from Elsevier.

Although serological assays are instrumental in diagnosing past infections, diagnosing diseases in their early stages allows better prognosis and more efficient medical care. At an early disease stage, the concentration of specific biomarkers is often extremely low, necessitating highly sensitive systems to detect such minimal quantities. In a test of sensitivity, using an MMB-based “sandwich” assay (Figure 5), we detected and quantified human interleukin-8 (IL-8), a known biomarker of inflammation and several cancer types. Using concentrations of IL-8 increasing from 0 ng/L to 50,000 ng/L, we performed dose response tests in both buffer and plasma, and extracted the LoDs (0.04 and 0.08 ng/L, respectively) [11]. In comparison with the commonly used ELISA assay, the MMB-based assay had a 20-fold better LoD (0.08 ng/L vs. 1.5 ng/L) and a wider dynamic range (6-logs vs. 4-logs) (Figure 5) [11].

The LoD calculated for the MMB-based assay was equal to that reported for a Bio-Rad IL-8 kit using a Bio-Plex 200 flow cytometer [20]. Compared to a flow cytometer, the MMB system is simpler to use, does not require a sophisticated fluidic system, and has a shorter processing time. The detection limits achieved using MMB are below the reported values in blood samples of healthy individuals, making it a relevant technique for use in clinical assays.

### 3.2. Detection of Interactions

Proteins often interact with other molecules, such as other proteins, DNA, and RNA, to carry out increasingly complex functions, such as delivering messages, protecting cells from invaders, enabling chemical reactions, transporting and storing other molecules (e.g., oxygen), providing mechanical support, and more. Malfunctioning protein–protein interactions (PPIs) and protein–DNA interactions are associated with various human diseases [26,27,28,29,30]. Thus, the interaction (or lack thereof) between proteins or between proteins and DNA sequences is an indicator of a disease or abnormal functioning of the organism.

Advanced techniques can identify these interactions, e.g., surface plasmon resonance-based techniques and microscale thermophoresis. In addition to detection and identification of interactions, these techniques enable kinetic study [31,32,33]. Nevertheless, due to their simplicity, the older classical biochemical methods—co-immunoprecipitation (Co-IP) coupled with Western blot for protein–protein interactions (PPIs) [34], and electrophoretic mobility shift assay (EMSA) for protein–DNA interactions [35]—are still regarded as gold standards. Both Co-IP and EMSA share some steps and features (e.g., a multi-step protocol, including running the samples on a gel, transferring them to a blot, and multiple washing steps). This protocol is laborious, prone to mistakes along the way, and time consuming (~4–72 h). In addition, for quantitative results and K_D_ extraction, densitometry analysis of the resulting bands is required. Such analysis is open to subjective user interpretation. Other challenges include non-specific binding, false-negative and false-positive results, and limited dynamic range [35,36,37].

**Figure 6:**
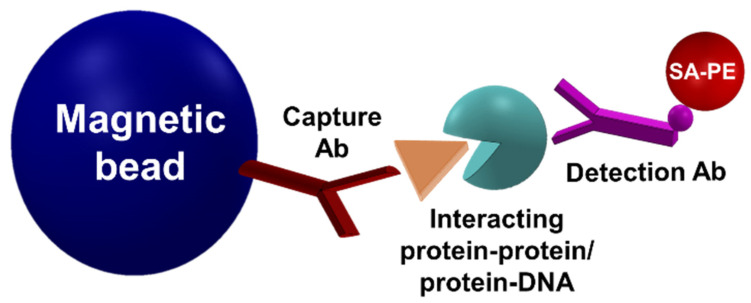
Simple representation of an MMB-based protein interaction detection assay.

MMB-based protein interaction detection assays overcome these challenges. Two assays have been developed, one to identify PPIs [16] and the other to identify protein–DNA interactions [38]. In both assays, magnetic beads are coupled with one of the interacting elements (protein/DNA), and a fluorescent molecule is coupled with the other element (protein/DNA). The magnetic beads and the fluorescent molecules are linked only when binding occurs, enabling the detection of the interaction (Figure 6).

**Figure 7:**
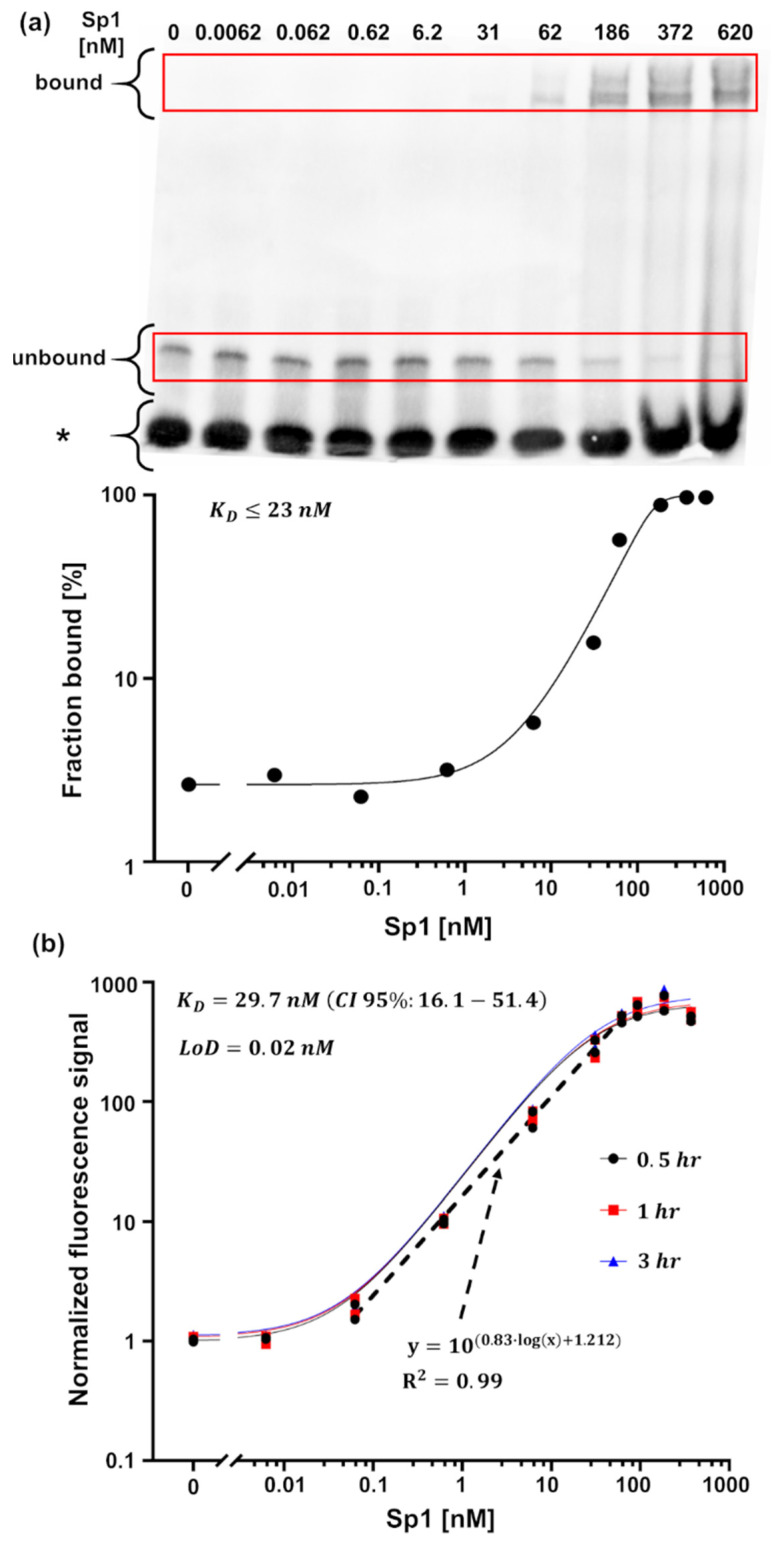
Dose response behavior of recombinant human Sp1 interaction with DNA. (**a**) A representative EMSA blot. The graph below the blot is the fraction of DNA bound with increasing concentrations of Sp1, calculated as (bound)/(bound+unbound). The band designated with an asterisk (“*”) represents the population of dsDNA that the protein is unable to bind. (**b**) Dose response at different incubation times (t) using the MMB system (n=2). Reprinted (adapted) with permission from [38] Copyright (2022) by American Chemical Society.

The MMB system has identified the known interactions between both recombinant and native erythropoietin (EPO) and erythropoietin receptor (EPOR) proteins [16]. It also identified the known interaction between a short (19 base pairs) GC-rich DNA sequence and both a purified specificity protein 1 (Sp1) and an overexpressed Buttonhead (BTD) protein in a cell lysate [38]. In both cases, the dissociation constant—a hallmark of quantitative interaction-detecting assays—was extracted. Using recombinant EPO and EPOR proteins, the calculated dissociation constant was $K_{D}=1.4\;\mathrm{nM}$, which corresponds to the reported high-affinity binding site of EPO [39]. In the case of Sp1, the extracted dissociation constant was $K_{D}=29.7\;\mathrm{nM}$, similar to the value extracted using EMSA ($K_{D}\leq 23\;\mathrm{nM}$) (Figure 7). Overall, using the MMB-based assays, the limits of detection (LoD) were on par with or lower than the concentrations detected using conventional methods. For example, compared with EMSA, the LoD of the MMB-based protein-DNA detection assay was ~310 times lower (0.02 nM vs. 6.2 nM) and the dynamic range was larger (4-log vs. 3-log) [38].

The modulation of the magnetic beads in the MMB system reduces the number of washing steps required for MMB-based PPI and protein-DNA assays, and consequently shortens the overall turnaround time to ~2 h. Moreover, coupling the magnetic beads with antibodies or proteins requires fewer reagents (e.g., 330 times less for PPI detection). In addition, in both assays, the non-specific binding to the magnetic beads was minimal.

In protein-DNA interactions, it is important to know whether the interaction is specific for a certain DNA sequence. To that end, a competition assay using mutated DNA sequences as competitors is usually done [40]. Using two mutated DNA sequences, we demonstrated through competition experiments that the MMB assay can assess the specificity of an interaction.

**Figure 8:**
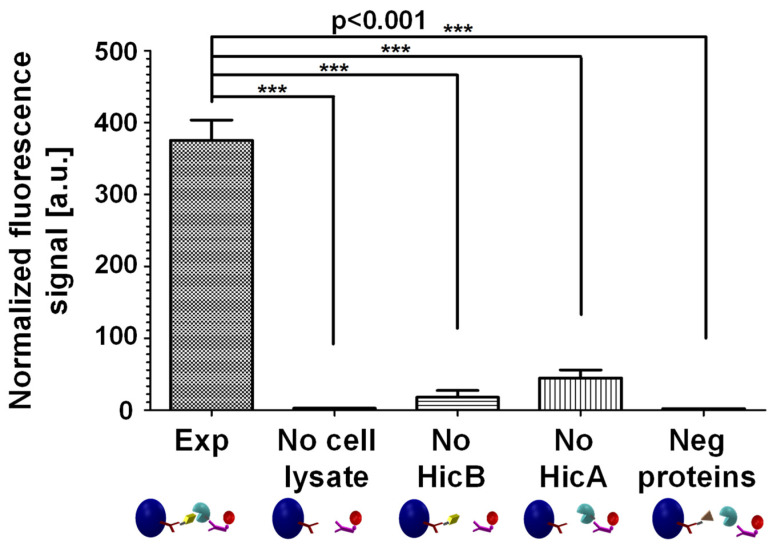
Identification of a novel toxin antitoxin pair, HicA and HicB, in *Pseudomonas aeruginosa*. Abbreviations: “Exp”, the experiment with cell lysate of *Pseudomonas aeruginosa* (*P. aeruginosa*) overexpressing HicA and HicB. “No cell lysate”, the same experiment without cell lysate. “No HicB”, the same experiment with cell lysate of *P. aeruginosa* overexpressing HicA solely. “No HicA”, the same experiment with cell lysate of *P. aeruginosa* overexpressing HicB solely. “Neg proteins”, the same experiment with cell lysate of *P. aeruginosa* overexpressing two proteins that are not known to interact with one another (SadB and ParD). Three asterisks (***) indicate a statistical significance of *p* < 0.001. Reprinted from [16], Copyright (2020), with permission from Elsevier.

In addition to verifying known interactions, the MMB-based PPI assay can also identify new interactions between proteins. Using *P. aeruginosa* as the model organism, we demonstrated that two proteins, HicA and HicB, form a protein–protein complex in a prokaryotic bacterial system (Figure 8). These two proteins act as a toxin–antitoxin system and thus can serve as a PPI target for antimicrobial treatment.

Compared to other commonly used methods, the MMB-based PPI and protein–DNA detection assays are less likely to produce false-positive results, for a couple of reasons. First, unlike Western blot, in which an artifact signal can originate from protein leftovers, in an MMB-based PPI assay, the oscillation of the magnetic beads ensures that only proteins that are attached to the magnetic beads contribute to the signal. Second, unlike fluorescence resonance energy transfer (FRET)-based methods, where a false signal can be produced by non-interacting proteins as a result of the proximity of their fluorescent labels, in an MMB-based PPI assay, the signal depends on the physical interaction between the proteins, not the proximity of their fluorescent labels [16]. In addition, unlike EMSA, the samples in the MMB-based assays are not run on a gel, and therefore the interactions are less prone to dissociate and produce false-negative results.

Many protein interactions contribute to the initiation or progression of disease, and they are all potential drug targets. As described below, the MMB-based assay has been applied for inhibitor screening of protein interactions, which is the first step in drug development.

#### Inhibitor Screening Using the MMB-Based Protein Interaction Assay

**Figure 9:**
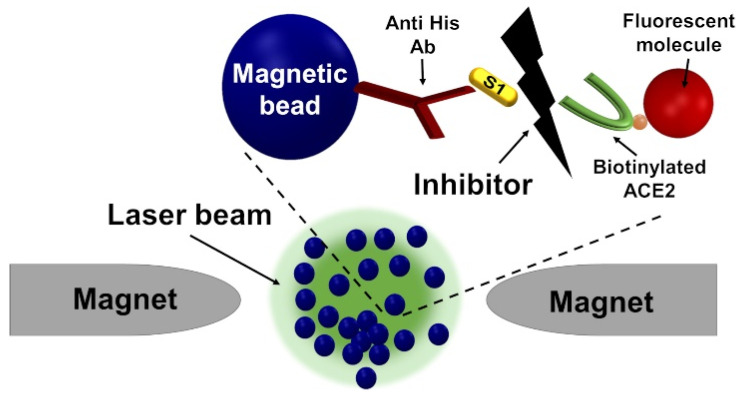
Schematic of using the MMB system for screening inhibitors of the S1-ACE2 interaction in SARS-CoV-2. From [41] with permission of MDPI 2021.

The recent outbreak of coronavirus emphasized the need for sensitive and rapid screening of inhibitors. We demonstrated that our assay can be applied for this purpose by screening potential inhibitors to the S1-ACE2 interaction in SARS-CoV-2, including neutralizing antibodies and small molecules (Figure 9).

Despite the inhibitory effect of the small molecule SSAA09E2 on the interaction in original SARS [42,43], we found that it is not an inhibitor to the S1-ACE2 interaction. However, we identified that the neutralizing antibody anti-S1 (clone 414-1) is an inhibitor for the interaction, with a half maximal inhibitory concentration (IC50) of 8.13 nM (CI 95%: 6.76–9.79). This value is on par with the results achieved by Active Motif (Carlsbad, CA, USA) in a neutralization test (15.77 nM) [44] and by Wan et al. in both neutralization and ELISA tests (1.75 nM and 2.96 nM, respectively) [45]. Although we achieved an LoD similar to those reported for commercially available ELISA kits (1.6 ng/mL), the dynamic range was much higher (4-log vs. 2-log) [46,47]. This improvement probably reflects the fact that the signal in an enzymatic ELISA reaches saturation faster than in fluorescence-based quantitative assays. 

Overall, the MMB-based PPI assay can detect and classify different types of molecules as inhibitors or non-inhibitors of the S1-ACE2 interaction, and it can be easily adapted to screen inhibitors of other PPIs.

### 3.3. Detection of Specific Nucleic Acid Sequences

Nucleic acids can also serve as biomarkers in multiple research and diagnostic assays. Detection of specific nucleic acid sequences and identification of subtle changes (mutations) in vitally important areas of the genome can be a warning sign of various diseases, either existing or in early stages of development. However, the concentrations of specific nucleic acid sequences in clinical samples (e.g., blood, saliva, urine) is usually low. Current nucleic acid detection methods include advanced and sensitive microscopy techniques, such as fluorescent in situ hybridization (FISH) [48], enzymatic amplification of the signal (e.g., enzyme-linked immunosorbent assay—ELISA) [12], and target amplification by quantitative polymerase chain reaction (qPCR) [48]. These detection methods have high sensitivity and specificity, but their prolonged and complicated testing protocols limit their diagnostic applicability. MMB-based molecular assays shorten and simplify these protocols, without compromising sensitivity and specificity. Generally, the abundance of the nucleic acid targets in the tested matrices and the presence of the potentially interfering substances dictate which MMB-based molecular assay should be used.

#### 3.3.1. MMB-Assisted Sandwich Hybridization Assay (SHA)

**Figure 10:**
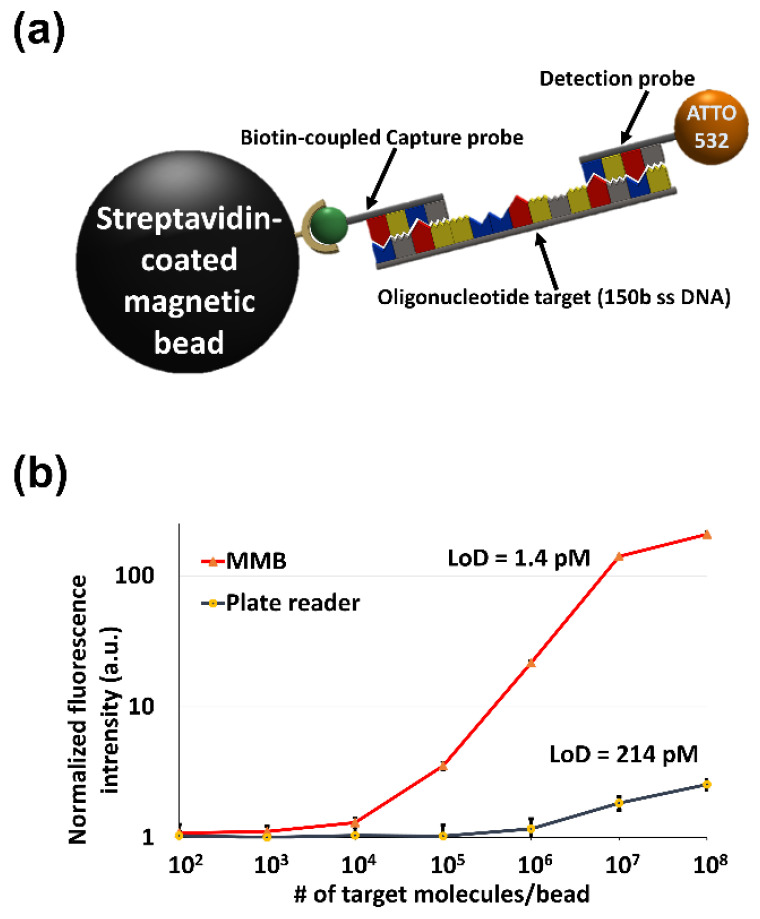
(**a**) Schematic of the sandwich hybridization assay (SHA). Capture and detection probes are hybridized to the target oligonucleotide and captured by streptavidin-coated magnetic beads. (**b**) Results of an SHA dose-response experiment performed with the MMB device (solid red line) and with a conventional fluorescence plate reader (solid black line). MMB achieves 150 times higher sensitivity than the plate reader. Reprinted from [18], Copyright © 2019 John Wiley & Sons—Books.

In the past three decades, PCR, which amplifies the sequence of interest to a detectable level, has become the gold standard for the detection of specific nucleic acid targets. However, in some cases the nucleic acid sequences cannot be directly amplified from the sample [49,50,51] and require additional extraction and purification. These steps are laborious and time-consuming, with relatively high costs. The sandwich hybridization assay (SHA) can mitigate these problems to a certain extent. Here, simultaneous hybridization of two oligonucleotide probes, complementary to the specific sites of the target molecule, results in creation of the “sandwich” (Figure 10a), which can be detected either electrochemically [52] or optically [53]. In PCR, the enzyme-based amplification is affected by the presence of PCR inhibitors. However, in SHA, no amplification is required, and therefore the hybridization of the probes can be performed on crude cellular extracts [53] without lengthy and laborious DNA/RNA extraction and purification steps. The target identification principle of the SHA is similar to PCR, providing comparable levels of specificity. The lack of amplification, however, can present a challenge for the conventional detection systems, due to the relatively low signal of the assay, requiring a highly sensitive detection system.

Using a sandwich hybridization assay, the MMB system detected as little as 1.4 pM of the target sequence (Figure 10b), which is ~150 times better than the LoD of a conventional fluorescence plate reader (~214 pM) [18]. The assay also tolerated the presence of point mutations in the target DNA sequence. Single mismatches had little to no effect on the assay’s sensitivity, while multiple mismatches significantly reduced the signal intensity, benefitting the assay’s specificity [18].

The sensitivity of the MMB-based SHA opens new possibilities for direct detection of specific oligonucleotide targets. For example, in industrial and environmental microbiology, as well as in molecular diagnostics, samples are frequently contaminated with PCR inhibitors. Two other potential application areas are forensics and paleontology, where DNA is frequently damaged by the environment [50,51], effectively preventing its direct amplification by conventional methods.

#### 3.3.2. Detection of Repetitive Nucleic Acid Sequences

Repetitive nucleic acid sequences are frequently found in the genomes of almost all living species [54,55,56]. The number of repetitions per genome ranges from hundreds to tens of thousands, and therefore detecting a sequence of interest requires only a minimum number of PCR amplification cycles, significantly reducing the testing time. Hence, repetitive sequences are the preferred targets for a variety of biomedical assays, but the relatively low optical sensitivity of mainstream detection systems (e.g., qPCR), combined with the high fluorescence background signal of conventional FRET-based probes, reduces the overall sensitivity of these tests and results in long testing times.

One field that can benefit from a fast and sensitive method for detecting repetitive DNA sequences is agriculture, specifically the laying-hen industry. Because only female chicks are required, male chicks are sorted out and killed on the day of hatching [57]. This ethical problem [58] can be resolved by determining the chick’s sex in ovo in the early stages of embryonic development and then disposing of unhatched eggs containing male embryos. The development of rapid methods for chick sexing is constrained by the need to perform thousands to hundreds of thousands of tests per day in an average hatchery, all quickly and with minimal costs.

**Figure 11:**
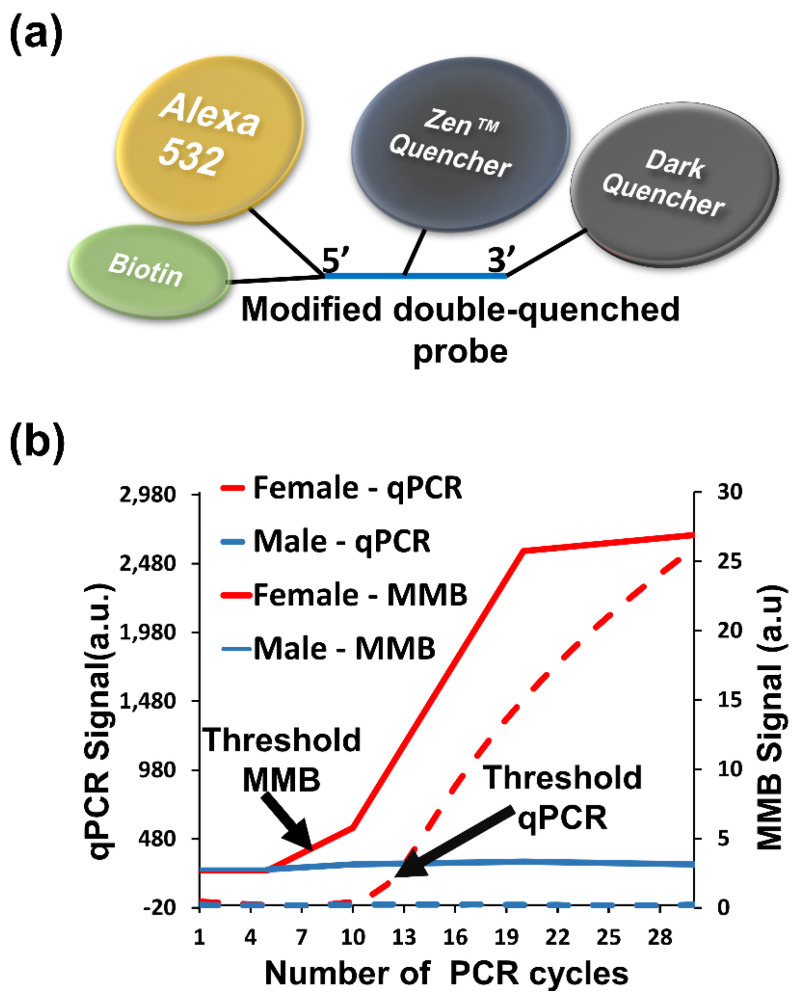
(**a**) Schematic of a modified double-quenched probe. (**b**) Comparative signal strengths in chick sexing experiments fusing the MMB system and qPCR. Using MMB, the sex of the hatchling could be determined after ~13 min, compared to 1–2 h using qPCR. Reprinted from [59], Copyright © 2019 American Chemical Society. Further permissions related to the material excerpted should be directed to ACS.

The MMB system, used with a modified double quenched probe (Figure 11a), can rapidly detect repetitive DNA sequences, and determine the chick sex in ovo in a timely manner. In a standard FRET-based probe, the long distance between the fluorophore and the quencher results in a highly fluorescent background for the intact probe. In a double-quenched probe, an additional quencher (e.g., Zen™ quencher) is positioned close to the reporter fluorophore and reduces the fluorescence background of the intact probe by ~90% [59]. In a modified double quenched probe (Figure 11a), a biotin molecule is connected to the same nucleotide as the reporter fluorophore. Thus, following PCR amplification, the free-floating fluorophores are still connected to the biotin molecules and can be collected by streptavidin-coupled magnetic beads. The significantly enhanced detection sensitivity of the MMB system enables the use of fewer amplification cycles than in a conventional qPCR system.

Using the modified double quenched probe and the MMB system, the female-specific repetitive XhoI sequence of the domestic chicken (*Gallus gallus*) was detected after only eight amplification cycles (Figure 11b) [59]. Consequently, the time required for sex identification of the embryo in ovo, with 100% specificity and sensitivity, was ~13 min, which is 4–9 times shorter than the time required for the same task with conventional qPCR, in which the results are available only after completion of the entire amplification process [59].

This research demonstrates that the time for detection of specific nucleic acid targets can be significantly reduced in comparison to mainstream detection methods. MMB-based DNA/RNA detection can also be successfully applied in other fields, including biomedical research and medical diagnostics.

#### 3.3.3. Detecting Low Abundance Nucleic Acid Targets

The recent COVID-19 pandemic has emphasized the importance of fast and sensitive methods for detecting low abundance nucleic acid targets (e.g., functional and structural genes of SARS-CoV-2). Unlike the repetitive nucleic acid sequences of complex organisms, there is only one copy of each target in the SARS-CoV-2 genome. In addition, to prevent the spread of the disease, it is important to diagnose it at the earliest possible stages, where the viral load is extremely low. Thus, 45 amplification cycles are usually employed in the gold-standard RT-qPCR tests and the time-to-result is ~90–120 min.

**Figure 12:**
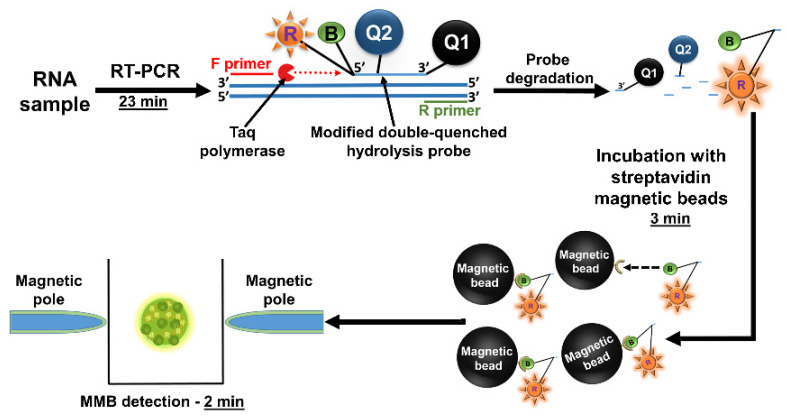
Workflow of the MMB-based SARS-CoV-2 detection assay. The turnover time from sample to result is approximately 30 min. Reprinted from [60], Copyright (2021) with permission from Elsevier.

Using a modified double-quenched hydrolysis probe, we developed an MMB-based molecular assay for rapid (<30 min) detection of SARS-CoV-2 nucleic acid targets (Figure 12) [60].

**Figure 13:**
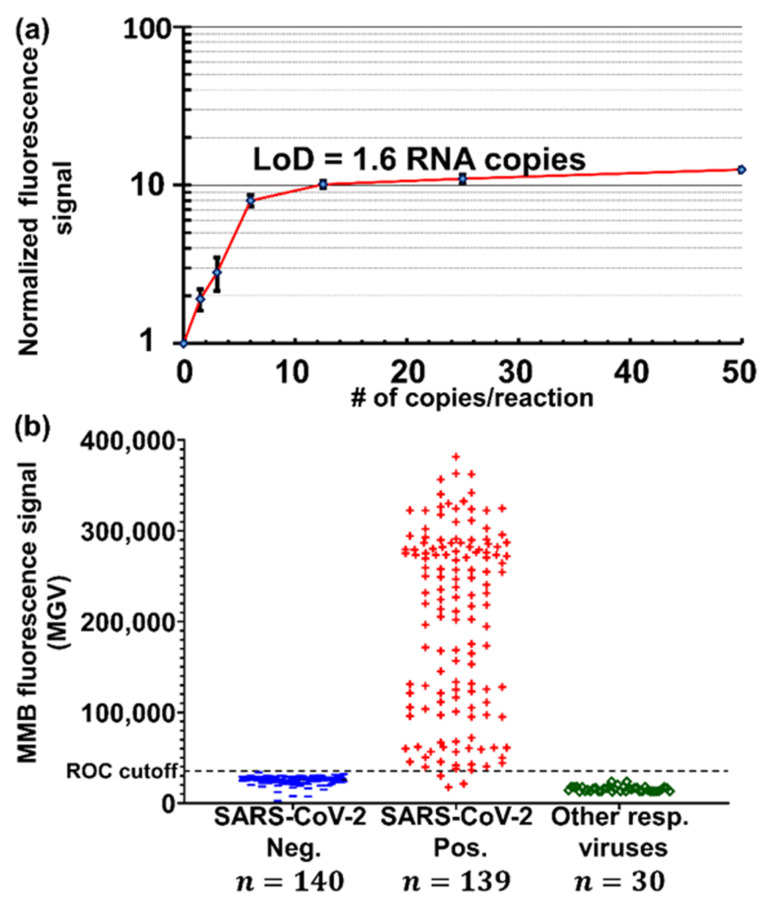
(**a**) Analytical performance of the MMB-based SARS-CoV-2 molecular assay. (**b**) Clinical performance of the MMB-based SARS-CoV-2 molecular assay. Reprinted (modified) from [60], Copyright (2021) with permission from Elsevier.

The short turnaround time of the assay is achieved by reducing the number of amplification cycles to 40 and reducing the duration of each amplification cycle to 23 s (compared to 90 s in regular RT-qPCR). Such a significant time reduction leads to suboptimal Taq polymerase performance and subsequently to a lower fluorescent signal. Normally, these conditions reduce the sensitivity of the assay. However, attaching magnetic beads to the fluorescent molecules and concentrating them to the detection volume significantly improves the optical detection sensitivity of the MMB system and compensates for the loss of signal. In addition, the end-point detection (the signal is read only at the end of the amplification process) allows further reduction of the turnaround time. To produce a dose response curve for the MMB-based SARS-CoV-2 molecular assay, we used in vitro transcribed SARS-CoV-2 targets (Figure 13a). The calculated LoD was 1.6 target copies per reaction, which is equivalent to 0.32 RNA copies/µL [60].

The MMB-based molecular assay was validated by blind-testing 309 clinical samples, of which 139 were collected from verified COVID-19 patients and 170 from SARS-CoV-2-negative individuals. In addition, 30 of the negative samples were positive for other respiratory viruses, such as influenza A, influenza B, or RSV. In clinical samples with verified RT-qPCR threshold values (Ct) of 37 and below (the relevant clinical range), the sensitivity and the specificity of the assay were both 100%, with 0% cross-reactivity with other respiratory viruses (Figure 13b). This level of clinical performance is identical to the performance of the “gold standard” RT-qPCR, but is achieved in one-third the time, allowing much higher throughput and, potentially, a higher degree of automation for clinical diagnostic labs.

## 4. Technology Advancements

**Figure 14:**
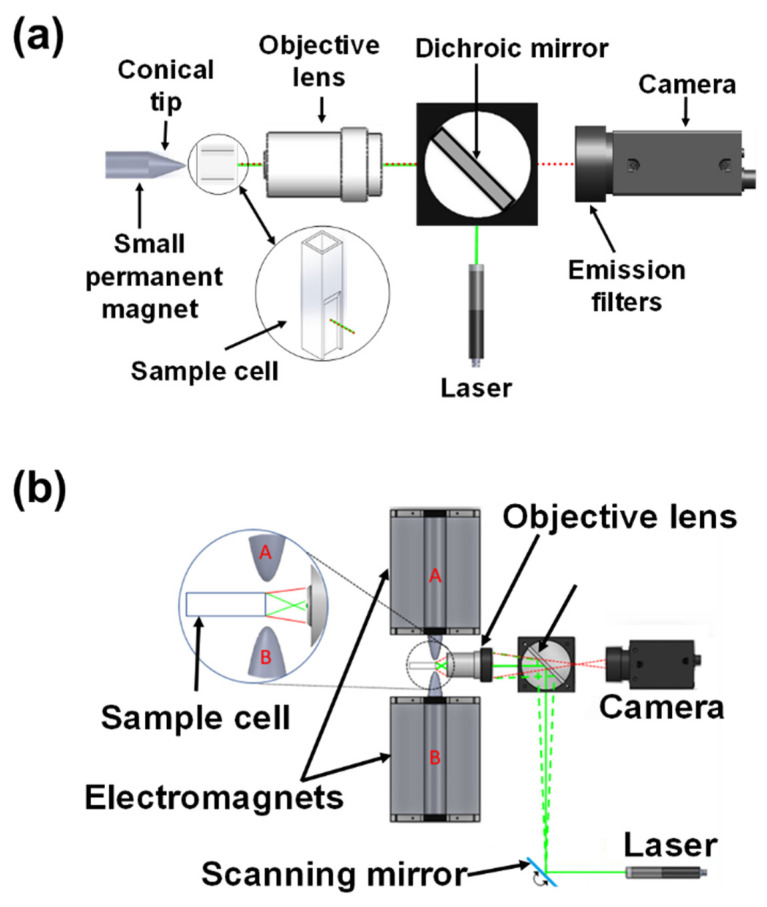
Schematic representations of (**a**) a magnetically aggregated biosensors (MAB) system. Reprinted from [15], with the permission of AIP Publishing 2019. (**b**) an optical modulation biosensing (OMB) system. Reprinted with permission from [61] © 2021 The Optical Society.

While MMB technology is highly sensitive and rapid, its relatively large electromagnets increase the bulk of the device. Here we describe two improvements to the original MMB platform: magnetically aggregated biosensors (MAB) and optical modulation biosensing (OMB) (Figure 14).

### 4.1. Magnetically Aggregated Biosensors (MAB)

A magnetically aggregated biosensor (MAB) system is more compact than an MMB detection platform [15]. Like MMB, in MAB the amplification of the signal is based on the aggregation of the magnetic beads into the detection volume. However, in MAB, instead of the two bulky electromagnets, the beads are aggregated and immobilized by a small permanent magnet with a sharp tip, significantly reducing the systems’ footprint (Figure 14a). The MAB system uses the same 532 nm laser diode module as the MMB system, working at 0.25 mW. The laser beam is diverted by a dichroic mirror and focused by an infinity-corrected objective lens to a 150 µm diameter beam spot on a polystyrene semi-micro sample cell that contains the biological reagents. The emitted fluorescence is collected by the same objective lens, passes through the dichroic mirror and two emission filters, and is focused by a plano-convex lens onto a CMOS camera [15].

**Figure 15:**
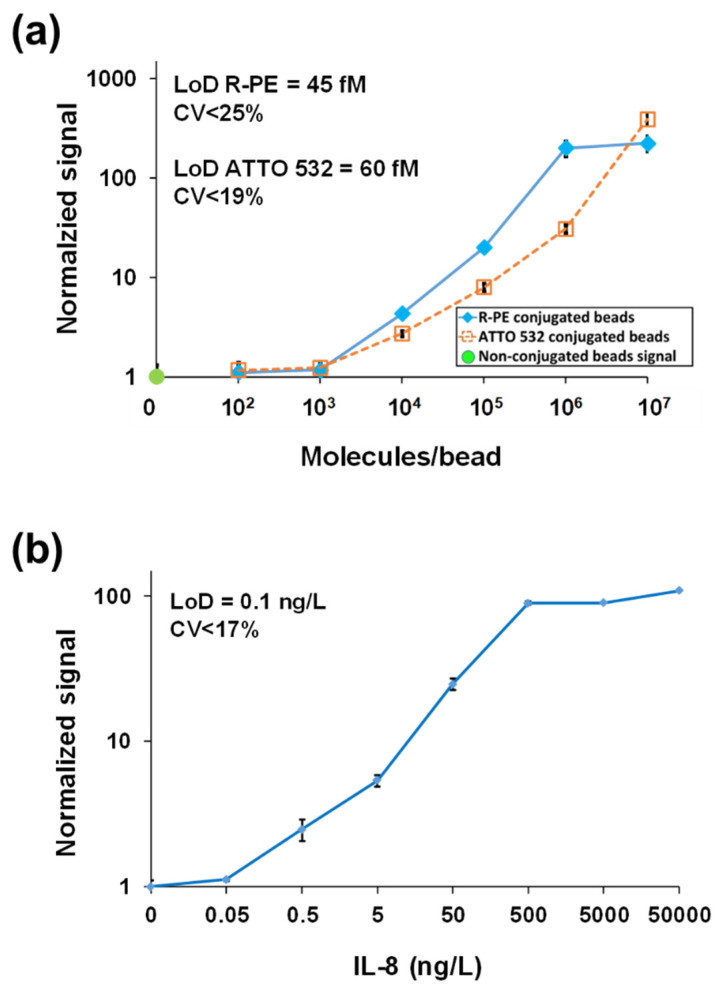
Dose-response experiments with the MAB system. (**a**) R-PE and ATTO 532 dose-response with M280SA magnetic beads. The calculated limit of detection (LoD) is 45 fM for R-PE and 60 fM for ATTO 532. (**b**) Recombinant human interleukin 8 (IL-8) dose-response (BioRad assay kit) in buffer. The calculated LoD is 0.1 ng/L. Reprinted from [15], with the permission of AIP Publishing 2019.

To demonstrate the analytical performance of the MAB system, we conjugated different concentrations of biotinylated R-PE and ATTO 532 fluorescent dyes directly to magnetic beads. The calculated limits of detection (LoD) for R-PE and ATTO 532 were 45 fM and 60 fM, with a 4-log dynamic range (Figure 15a). To evaluate the MAB system’s performance in a simulated clinical immunoassay, recombinant human interleukin 8 (IL-8) was used with a commercially available IL-8 assay kit (BioRad, CXCL 171BK31MR2). The calculated LoD of the MAB-based human IL-8 assay in buffer was 0.1 ng/L (Figure 15b). Overall, the sensitivity achieved with the MAB system in controlled laboratory experiments was lower than that of the conventional MMB device [11,15], but still comparable to the sensitivity of current state-of-the-art laboratory assays [62]. In MAB, the slightly lower sensitivity is compensated for by the significantly smaller dimensions and reduced power consumption of the system.

In MAB, because the cluster of beads is constantly held in the laser beam, the variability of the observed signal is low. Therefore, the required number of acquired images is much lower than in the MMB system (3 vs. 600), and consequently the data acquisition time is much shorter (0.06 s vs. 12 s) [15]. However, the drawback of collecting the signal from a single spot in the center of the bead cluster is the lack of a reference reading from the background. Hence, to remove background noise from unbound fluorescent molecules, the MAB-based assay includes several washing and separation steps. In addition, in the MMB system, the magnetic beads are physically manipulated from side to side, in and out of the laser beam. Thus, every time the beads enter the laser beam a different area of the beads’ surface faces the excitation beam and the number of fluorescent molecules per beads can be averaged over multiple images. In MAB, the beads are collected once and then their collective fluorescence signal is measured. Thus, inhomogeneous coverage of the beads’ surface with fluorescent molecules may introduce inaccuracy to the measurement, particularly at low concentrations of the target analyte [15].

### 4.2. Optical Modulation Biosensing (OMB)

The recently introduced optical modulation system (OMB) integrates the operating principles of the MMB (i.e., measuring the signal from both the beads and the background) and the MAB systems (i.e., fixing the cluster of beads in a single spot) [61]. In OMB, the beads are immobilized to one side of the sample holder and the relative movement between the beads and the laser beam is achieved by manipulating the laser beam from side to side. Thus, the laser beam alternatively illuminates the aggregated beads on one side of the cuvette and the background solution on the other side.

Similar to the MMB system, the OMB system (Figure 14b) uses a 532 nm laser diode, working at 0.25 mW. The laser beam is moved laterally back and forth by a scanning galvo mirror and deflected by a dichroic mirror into a microscope objective lens [61]. The objective lens focuses the beam to a 150 µm diameter spot on a sample cell containing the magnetic beads. The same objective lens collects the emitted fluorescence and passes it back through the dichroic mirror and two emission filters. The fluorescence is then detected by a CMOS camera. In the OMB system, the beads are first aggregated by two electromagnets working in alternation and then held to one side of the sample cell by activating one of the electromagnets while deactivating the second one.

**Figure 16:**
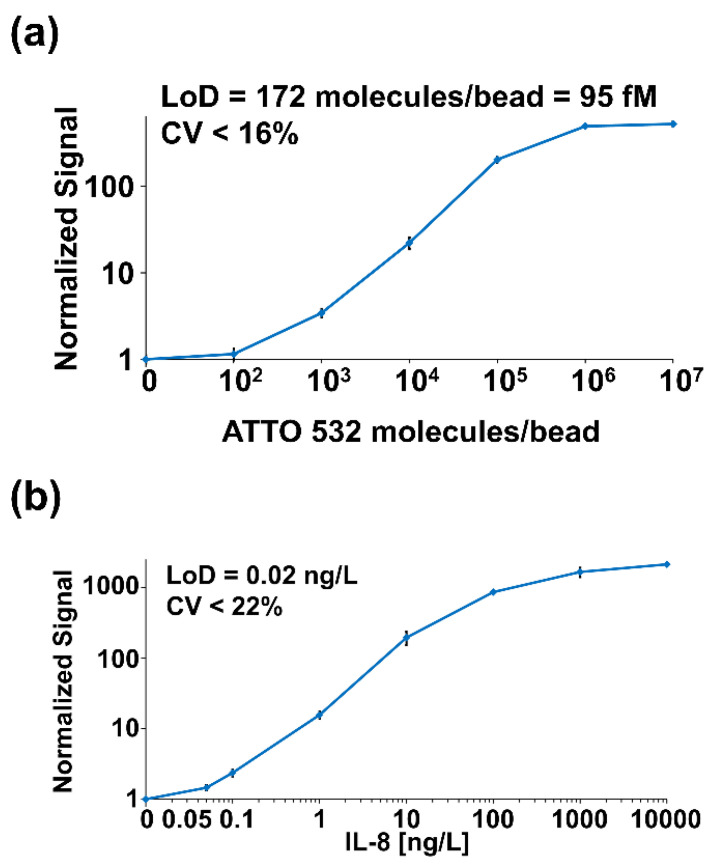
Dose-response experiments with the OMB system. (**a**) ATTO 532 dose-response with streptavidin-coupled M280 magnetic beads. The calculated limit of detection (LoD) is 95 fM, and the coefficient of variation (CV) is less than 16%. (**b**) Recombinant human interleukin 8 (IL-8) dose-response in buffer. The calculated LoD is 0.02 ng/L. Reprinted with permission from [61] © 2021 The Optical Society.

To demonstrate the analytical performance of the OMB system, we conjugated different concentrations of biotinylated ATTO 532 fluorescent dye to streptavidin-coupled magnetic beads. The calculated LoD was 95 fM (Figure 16a). To further evaluate the OMB system’s performance in a simulated clinical immunoassay (Figure 16b), recombinant human IL-8 was used with a commercially available IL-8 assay kit. The calculated LoD of the OMB-based human IL-8 assay in buffer was 0.02 ng/L [61]. This LoD is comparable with the LoD (0.04 ng/L) achieved by the MMB system for the same assay [11].

Modulating the laser beam is much faster than physically manipulating the magnetic beads. Therefore, acquiring images of the illuminated beads and the background can be done in a fraction of a second, limited only by the frame rate of the camera. Overall, the data acquisition time of the OMB system is ~250 milliseconds, much shorter than the ~12 s of the MMB system [15].

A summary of all the MMB applications and the reported parameters can be found in Table 1.

## 5. Discussion

The latest advances in medical diagnostics and biomedical research would not be possible without technologies that offer rapid and highly sensitive detection of a wide range of biomolecules. However, many of the existing detection systems suffer from high cost, bulk, and complicated working protocols.

Here, we reviewed different applications of the novel MMB system and demonstrated its rapid turnaround time, high sensitivity, and ease of use. In MMB, the signal amplification is achieved by attaching magnetic beads to the fluorescently labeled target molecules, aggregating them from the entire sample into the detection area, and then separating the signal from the background noise by modulation. Compared with gold standard detection methods, MMB offers better sensitivity, shorter assays, and ease of use in a compact and affordable format that does not require frequent and costly calibration.

It should be noted that aggregating all the beads into a small detection volume improves the sensitivity but limits the number of analytes that can be simultaneously detected in a single sample cell. Hence, the primary purpose of the MMB system and its modifications is to rapidly provide the highest possible sensitivity, which compensates for the limited multiplexing capability.

There are several factors affecting the selection of magnetic beads for the experiments. First, the magnetic force acting on the magnetic beads depends on both the magnetic field gradient of the magnetic poles and the beads’ magnetic saturation moment [17]. Thus, to rapidly aggregate the beads and shorten the detection time, the magnetic beads used in this research have high magnetic saturation moment. Second, to avoid self-aggregation of the beads when the external magnetic field is turned on and off, the beads should be superparamagnetic. Third, to reduce background noise, it is best to use magnetic beads with low auto-fluorescence [63].

The MMB system is relatively compact, with the potential for further miniaturization. By replacing the relatively bulky electromagnets with a small permanent magnet, the recently introduced MAB system reduces the overall dimensions of the original MMB system, but it adds washing and separation steps to the assay’s protocol. By manipulating the laser beam rather than the magnetic beads, the OMB system provides high sensitivity, while keeping the wash-less protocol and shortening the data acquisition time. Future work that combines optical beam modulation with the use of a permanent magnet will enable rapid detection of target molecules in a conventional 96-well plate.

Current research and development efforts are focused on improving the potential throughput of the devices by enabling simultaneous rapid readout from multiple wells. To do that, an optical system capable of splitting the laser beam into multiple beams has to be developed. Future research directions will also include the development of multiplexing capabilities for simultaneous detection of two or more analytes in a single well.

Another research direction is related to the recent advances in microfluidics and, specifically, in “lab-on-a-chip” technology, which opens new possibilities for development of the point-of-care diagnostic methods. This technology reduces the assays hands-on time by automatically performing multiple sample preparation steps in a single disposable microfluidic cartridge. Adapting the MMB/OMB systems to work with the “lab-on-a-chip” disposable cartridges will allow development of the highly sensitive MMB/OMB-based diagnostic assays for the point-of-care applications.

Overall, MMB technology is very robust and can be used in a wide variety of applications, including detection of specific nucleic acids, proteins, antibodies, and protein interactions. Moreover, the presented applications can be easily adapted to investigate other interactions and biomarkers. The recently demonstrated MMB-based method for rapid molecular diagnosis of SARS-CoV-2 opens new possibilities and allows development of tests for rapid detection of other pathogens, such as dengue, West Nile, and many others. These viruses are considered emerging pathogens worldwide, and the demand for a fast and reliable detection method is extremely high.

## Figures and Tables

**Table 1:** Summary of different applications using the MMB and its advancements.

	Application	System	Target	Analytical Parameters		Clinical Parameters	
				LoD	Dynamic Range	Sensitivity [%]	Specificity [%]
Protein assays	SARS-CoV-2 serological assay	MMB	anti-SARS-CoV-2 S1 IgG	129 ng/L	4-log	93	98
	ZIKV serological assay	MMB	anti-ZIKV NS1 IgM	99 ng/L	4-log	88	100
	ZIKV serological assay	MMB	anti-ZIKV NS1 IgG	102 ng/L	4-log	97	100
	Detection of PPIs	MMB	EPO-EPOR interaction	42 ng/L	4-log	-	-
	Detection of PPIs	MMB	S1-ACE2 interaction	1600 ng/L	4-log	-	-
	Inhibition of PPIs	MMB	S1-ACE2 interaction	-	3-log	-	-
	Detection of protein-DNA interactions	MMB	Sp1-wt DNA interaction	1610 ng/L	4-log	-	-
	Detection of protein biomarkers	MMB	IL-8	0.08 ng/L	6-log	-	-
Molecular assays	Direct detection of specific DNA markers	MMB	EML4-ALK oncogenic translocation	65.2 ng/L	3-log	-	-
	Detection of the repetitive nucleic acid sequences	MMB	*XhoI* repetitive sequence of the female chicken	-	-	-	-
	Clinical diagnosis of SARS-CoV-2	MMB	E-gene	1.6 target copies/reaction	-	100	100
Technology advancements	Detection of protein biomarkers	MAB	IL-8	0.1 ng/L	4-log	-	-
	Detection of protein biomarkers	OMB	IL-8	0.02 ng/L	4-log	-	-
